# Development of a Closed Chest Model of Chronic Myocardial Infarction in Swine: Magnetic Resonance Imaging and Pathological Evaluation

**DOI:** 10.1155/2013/781762

**Published:** 2013-10-27

**Authors:** Verónica Crisóstomo, Juan Maestre, Manuel Maynar, Fei Sun, Claudia Báez-Díaz, Jesús Usón, Francisco M. Sánchez-Margallo

**Affiliations:** ^1^Jesús Usón Minimally Invasive Surgery Centre, Carretera N-521, Km 41.8, 10071 Cáceres, Spain; ^2^Las Palmas de Gran Canaria University (ULPGC), C/Juan de Quesada No. 30, 35001 Las Palmas de Gran Canaria, Spain

## Abstract

Our aim was to develop an easy-to-induce, reproducible, and low mortality clinically relevant closed-chest model of chronic myocardial infarction in swine using intracoronary ethanol and characterize its evolution using MRI and pathology. We injected 3-4 mL of 100% ethanol into the mid-LAD of anesthetized swine. Heart function and infarct size were assessed serially using MRI. Pigs were euthanized on days 7, 30, and 90 (*n* = 5 at each timepoint). Postoperative MRI revealed compromised contractility and decreased ejection fraction, from 53.8% ± 6.32% to 43.79% ± 7.72% (*P* = 0.001). These values remained lower than baseline thorough the followup (46.54% ± 11.12%, 44.48% ± 7.77%, and 40.48% ± 6.40%, resp., *P* < 0.05). Progressive remodeling was seen in all animals. Infarcted myocardium decreased on the first 30 days (from 18.09% ± 7.26% to 9.9% ± 5.68%) and then stabilized (10.2% ± 4.21%). Pathology revealed increasing collagen content and fibrous organization over time, with a rim of preserved endocardial cells. In conclusion, intracoronary ethanol administration in swine consistently results in infarction. The sustained compromise in heart function and myocardial thinning over time indicate that the model may be useful for the preclinical evaluation of and training in therapeutic approaches to heart failure.

## 1. Introduction

Cardiovascular diseases are a major cause of death and disability in developed countries. Myocardial infarction has an estimated annual incidence in the US of 525.000 new and 190.000 recurrent attacks. Approximately 15% of patients who suffer a coronary attack will die as a result of it [1]. Patients who survive a heart attack have an increased prevalence of heart failure (HF) [2].

Research in animal models is mandatory to assess safety and efficacy of any new therapy prior to clinical translation [3, 4]. Large animal models are essential to test new interventional, surgical, or electrophysiological procedures that cannot be performed in small animals, thus providing a platform for training specialists in optimal techniques involving new procedures, another highly important role of animal models of disease [5]. Current models for myocardial infarct creation are suboptimal for a variety of reasons, including a high mortality and inconsistent infarct creation [6–9]. 

Clinically, intracoronary ethanol has been used for the management of symptomatic hypertrophic obstructive cardiomyopathy (HOCM) [10, 11] and in the therapy of arrhythmias. Studies using intracoronary ethanol administration in animals are limited and generally focused on refining HOCM therapies [12] or electrophysiological procedures [13]. To our knowledge, the natural history and time course of intracoronary ethanol administration for myocardial infarction induction remain to be explored. A comprehensive evaluation of the evolution over time of any MI model using clinically validated and widely used techniques, such as MR imaging, could be highly useful for researchers when there is a need to select the best model for their experimental work. 

The aim of this study was to develop a simple, reproducible, clinically relevant, and low mortality endovascular model of chronic myocardial infarction in the swine. If successful, this model should be useful for preclinical evaluation of therapeutic approaches to HF after chronic myocardial infarction, such as regenerative therapies or electrophysiological studies, as well as for training in diagnostic and therapeutic interventions. 

## 2. Materials and Methods

The study protocol was approved by the Institutional Animal Care and Use Committee, and it complied fully with the Guide for the Care and Use of Laboratory Animals: Eighth Edition (National Research Council. Washington, DC: the National Academies Press, 2010). A total of 18 female domestic swine weighing 40.9 ± 10.48 kg were used for this study. Animals received 400 mg oral amiodarone and 500 mg aspirin from two days before to 7 days after infarct induction. Surviving animals were euthanized after infarct induction at day 7 (Group I, *n* = 5), day 30 (Group II, *n* = 5), and day 90 (Group III, *n* = 5). 

### 2.1. Anesthesia and Monitoring Protocol

All the procedures were performed under general inhalant anesthesia. After being fasted for 24 hours, animals were premedicated with a combination of diazepam (0.1 mg/kg) and ketamine (10 mg/kg) injected intramuscularly (IM). Anesthesia was induced with intravenous (IV) propofol (4 mg/kg). Endotracheal intubation was performed and anesthesia was maintained with sevoflurane administration (adjusted to a 3.3% End tidal sevoflurane). A bolus (5 g/kg) of fentanyl was administered every 30 minutes. Continuous infusion of lidocaine at a rate of 1 mg/kg/h was used until the end of anesthesia. Systemic heparin was injected IV (150 IU/kg) 5 minutes before percutaneous sheath placement. A fentanyl transdermic release patch was used to assure correct analgesia in the immediate postoperative period. Prophylactic antibiotics was administered in all cases for 5 days (ceftiofur hydrochloride). Electrocardiography (ECG) (Hewlett Packard model 86S, Hewlett Packard, Geneva, Switzerland) (lead II) and pulse oximetry with a probe (Clip Tip sensor, Oximeter Sensor, Datex-Ohmeda, Louisville, CO) placed on the tongue, rectal temperature, tidal volume, end-tidal concentration of sevoflurane, end-tidal CO_2_ concentration, and respiratory rate (Ohmeda RGM 5250, Ohmeda, Madrid, Spain) were monitored through the procedure. 

Blood samples were taken for troponin I assay immediately before infarct creation (baseline) and 2 hours, 24 hours, and 7 days after ethanol infusion. 

### 2.2. MR Examinations

Cardiac MR studies were performed for morphological and functional assessment, before infarct induction, immediately after and at 7, 30, and 90 days using a 1.5 T system (Intera 1.5 T, Philips Medical Systems. Best, The Netherlands). Baseline (before induction) examination was performed without gadolinium administration. A group of healthy swine (Control group), weighing the same as the study animals on days 30 (*n* = 5) and 90 (*n* = 5), were also imaged to evaluate growth-related changes in cardiac function. These animals had not been subjected to any intervention. Data obtained from these animals was compared to the values obtained from Groups II and III, in order to avoid the influence of the animals' growth on the interpretation of the evolution of cardiac function parameters. 

Images were acquired in the intrinsic cardiac planes: short axis, vertical long axis, and horizontal long axis views. For measurement of left ventricular function and mass breath hold gradient echo cine images were obtained over the entire left ventricle (LV) with the following parameters: slice thickness: 8 mm, no gap, field of view (FOV): 320 × 320 × 80, matrix: 192 × 192, flip angle: 60°, and repetition time/echo time (TR/TE): 4.4/2.2. The number of cardiac phases acquired per slice was 25. Infarct size was measured in images acquired 5 to 15 minutes after the injection of 0.2 mmol/kg of a gadolinium-based contrast agent (Gadobutrol) using a breath-hold 3D gradient-echo inversion-recovery sequence. Inversion time was chosen selecting the time that yielded the best nulling of the myocardial signal. Imaging parameters used for the delayed enhancement (DE) images were slice thickness: 8 mm, no gap, FOV: 330 × 330 × 50, matrix: 224 × 200, flip angle: 15°, and TR/TE: 4.9/1.67. MR images were analyzed to calculate the following LV parameters: end diastolic volume (EDV), end systolic volume (ESV), ejection fraction (EF), wall mass, cardiac output (CO), and percentage of infarcted LV.

### 2.3. Infarct Induction

**Figure 1:**
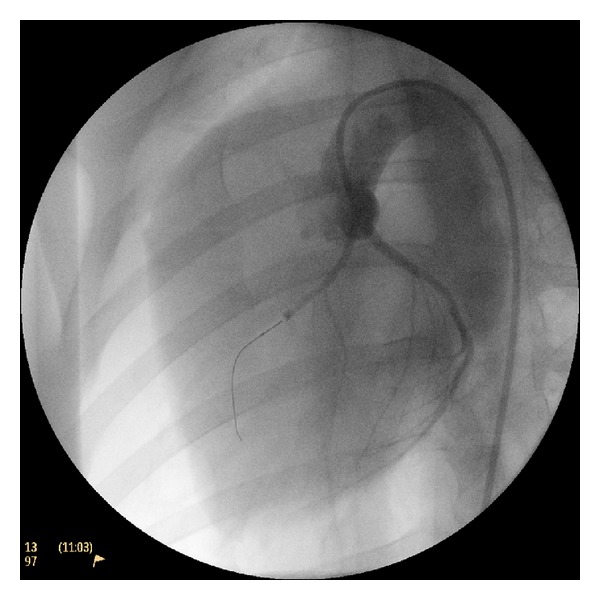
Coronary angiogram obtained immediately after balloon inflation to assess correct occlusion of the artery prior to ethanol injection.

Under sterile conditions, a right femoral arterial access was established using the Seldinger technique and a 7Fr introducer sheath (Terumo, Inc. Tokyo, Japan) was placed percutaneously. Under fluoroscopic guidance (Philips BV Pulsera, Philips Medical Systems, Best, The Netherlands) a 6Fr hockey stick guiding catheter (Mach 1, Boston Scientific Corporation, Natick, MA, USA) was introduced and placed at the origin of the left coronary artery. Coronary angiograms were obtained in the 40° left anterior oblique (LAO) projection to better demonstrate the length of the left anterior descending coronary artery (LAD), and a 0.014′′ guidewire (Hi-torque. Abbott Vascular, Santa Clara, CA, USA) was advanced inside this artery. An over-the-wire coronary balloon of appropriate diameter (typically 3 mm. Apex OTW, Boston Scientific Corporation, Natick, MA, USA) was advanced to the LAD and positioned either below the larger diagonal branch (first or second, if one of them was clearly larger) or, in those animals whose anatomy did not show a dominant diagonal, immediately below the second diagonal branch. The balloon was inflated and correct occlusion was assessed by contrast injection through the guiding catheter (Figure 1). The wire was then removed and the balloon lumen was flushed with 5 mL of saline before injecting 3 mL of absolute ethanol at a rate of 1 mL/min. A further 5 mL of heparinized saline was administered before balloon deflation and removal. A postprocedural coronary angiogram was then obtained. Animals were kept under anesthesia with lidocaine infusion for another hour, before being sent for an immediate MR follow-up study. 

Immediately after the last MR examination, animals were euthanized administering a lethal dose of potassium chloride while under general anesthesia. The explanted hearts were submerged in 4% formalin for a minimum of 48 hours and, once fixed, were cut into 5 mm thick slices. 

Samples were taken from the infarct and transition areas for pathological examination, embedded in paraffin, sliced into 5 *µ*m thick sections, and stained with hematoxylin and eosin and Gallego's trichromic. 

### 2.4. Statistics

Since the objectives of the study are descriptive in nature, no formal hypothesis testing was done. Data are presented as means and standard deviations. Comparisons between cardiac function parameters between groups were performed using the Mann-Whitney *U* test. Values of *P* < 0.05 were considered significant. All *P* values were the results of 2-tailed tests. Calculations were performed using the SPSS 15.0 statistical package for Windows (SPSS Inc., Chicago, Ill).

## 3. Results

Cardiac catheterization and ethanol injection were technically successful in all pigs. Changes in the ECG, such as premature ventricular complexes and ST segment elevation, were seen in all animals during ethanol injection. The ST wave remained elevated during the one-hour observation time in all cases. Seven pigs developed ventricular tachycardia and fibrillation after balloon deflation. Four pigs were successfully recovered using 200J biphasic defibrillation shocks and pharmacological therapy when needed, while the other 3 animals (16.6%) died during infarct creation. The remaining animals completed their allotted followup (no further deaths). 

Baseline troponin I levels were 0.037 ± 0.027 *µ*g/L. A significant (*P* < 0.005) increase in this parameter was seen at both 2 hours (7.43 ± 3.13 *µ*g/L) and 24 hours (27.5 ± 6.28 *µ*g/L) after ethanol injection. Troponin I levels measured 7 days after infarct induction had returned to baseline values (0.055 ± 0.029 *µ*g/L, *P* = 0.261), thus confirming acute MI [14]. 

**Figure 2:**
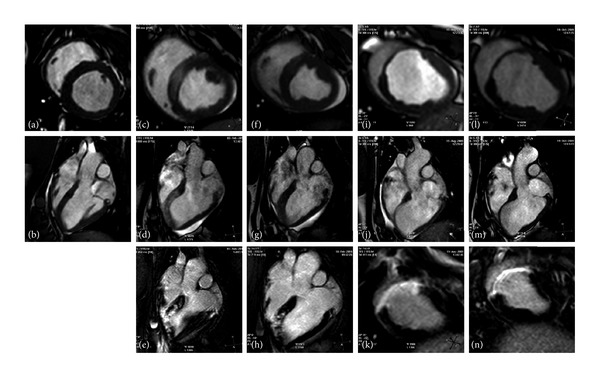
Cardiac MR images obtained during the study. Cine images obtained in short axis and four chamber views at all timepoints are shown in the first two rows. The third row depicts delayed enhanced acquisitions. (a) and (b) Baseline examinations showing normal swine cardiac anatomy. (c), (d) and (e) Images acquired immediately after infarct induction. The infarcted area can be identified by a density change in the cine images, which correspond to the enhanced area on the viability study. (f), (g) and (h) Images obtained 7 days after infarction. Little changes were evidenced in the cine images at this timepoint (apart from akinesia of the septum), but the DE view shows the typical hyperenhancement appearance of the infarct. (i), (j) and (k) Study performed on the 30-day followup. Wall thickness is clearly decreased in the infarcted area (anteroseptal), corresponding with the hyperenhanced septum in the viability study. (l), (m) and (n): 90-day followup. The infarcted area is clearly identified as a very thin wall in the cine acquisitions and as a highly enhanced area in the viability study.

Postoperative MR studies revealed a density change in the midanteroseptal and apical septal areas of the left ventricle (Figure 2), along with compromised contractility of the affected LV wall (see Supplemental Video in the Supplementary Material available online at http://dx.doi.org/10.1155/2013/781762). 

MR-derived cardiac function parameters are presented in Table 1. EF decreased significantly (*P* < 0.05), compared to baseline, in all studied timepoints, decreasing form 53.8% ± 6.32% to 43.79% ± 7.72% immediately after infarct induction. These values remained lower than baseline thorough the followup (46.54% ± 11.12%, 44.48% ± 7.77% and 40.48% ± 6.40%, resp., *P* < 0.05). Moreover, EF values from healthy controls were significantly (*P* < 0.05) higher than infarcted animals, measuring 54.04% ± 5.81% in the group weight-matched to the study animals at 30 days and 57.64% ± 8.48% in the animals weight-matched to the 90-day group. EDV and ESV were also significantly (*P* < 0.05) higher than baseline through the followup, except in the case of EDV measured 2 hours after infarct induction, where this difference did not reach statistical significance. Similarly, ventricular volumes measured from healthy controls were consistently lower than the corresponding data from the infarcted animals, but this difference was significant only in the ESV values obtained at 90 days. No changes were seen in cardiac output during followup. 

A clearly demarcated area of hyperenhancement comprising the midanterior and anteroseptal and apical septal left ventricle was visualized in all animals at the 7, 30, and 90-day follow-up MRI examinations. The size of the infarct, measured in percentage, decreased over time (Table 1). Progressive LV remodeling, with a decrease in thickness of the infarcted area, was seen in all animals at 30 and 90 days (Figure 2). 

Pathology on day 7 revealed a clearly defined area of coagulation necrosis comprising both small vascular structures and cardiac fibers (myocardiocytes that exhibited highly eosinophilic cytoplasms and no nuclei) with moderate hemorrhage and band composed by granulation tissue with some neovessels and a cellular infiltrate rich in macrophages, lymphocytes, and fibroblasts.

On day 30, there were still some necrotic areas within granulation tissue, but most of the necrotic tissue was completely replaced by the latter. This granulation tissue was more mature, showing a decrease in the cellular component, mostly composed by fibroblasts at this time, increased collagen content, and a significant decrease in neovessels. Occasional calcifications and bundles of apparently healthy conduction cells at the subendocardial level were seen. 

On day 90, injured areas exhibited a highly organized appearance with collagen fibers, consistent with fibrosis and scar formation. In transition areas, collagen fiber bundles were seen interspersed between normal myocardiocytes. A very thin wall could be seen in some areas showing preserved conduction cells near the endocardial border, some myocardiocytes groups and organized fibrous tissue.

## 4. Discussion

The ideal experimental model should meet a number of criteria so that the results can be extrapolated to the clinical setting [4, 6, 9, 15]. Such a model should (1) mimic the human disease as much as possible; (2) be reproducible; (3) allow control over the duration and location of the ischemia; (4) be performed via a minimally invasive approach, avoiding any possible additional influence on experiments in order to minimize variables that could influence outcomes; (5) require short preparation time, because the subsequent experiments are often time consuming; (6) allow easy experimental animal management; (7) cause significantly impaired cardiac function, and (8) be cost-effective. Cardiology subspecialties that benefit from the use of animal models are varied, and therefore it is important that the researchers involved are familiar with the different models available and, when deciding which one to use, select the one best suited to the intended use [3].

The model we present herein meets most of these requirements. Swine were selected for the present study because they have many similarities to the human heart in terms of cardiovascular anatomy, ventricular performance, cardiac metabolism, electrophysiology, coronary artery distribution, and collateralization after an acute event [16–19], thus allowing the creation of models representative of the human disease. All animals surviving the infarction procedure developed transmural infarcts of similar sizes at the same locations. The ethanol injection can be performed at any coronary level, in order to achieve different sizes of infarct. Preparation time is shorter than in most reported models mainly related to the noninvasive nature of the presented model and the short ethanol injection time, 3 to 4 minutes, when compared to occlusion models, that maintain the balloon inflated up to 150 min [9, 20]. Swine management is straightforward in the hands of an experienced team of anesthesiologists, technicians and veterinary surgeons. Impairment of cardiac function is moderate but sustained over time, allowing for longer term studies. In terms of cost, ethanol is a widely available and inexpensive compound. Moreover, the short time needed for MI induction translates into low anesthetic and staff costs, and the low mortality rates contribute to the model affordability. 

Generally, chronic infarctions in large animal models are obtained by surgical ligation of a coronary artery via a thoracotomy [19, 21–23], the use of the ameroid constrictor, or occlusion, either temporary using an angioplasty balloon catheter [6–9, 15, 17, 20, 24–29] or permanent with different embolization systems, including the endovascular deployment of a “flow reductor” inside the target coronary artery [30], microbead embolization [31, 32], embolization using gelfoam alone [4, 33] or in combination with coils, [34] and deployment of an ivalon plug [35]. A closed chest minimally invasive model is more comparable to human MI pathology than open chest models [27], and it carries less risk of complications and infections [30]. It is known that opening the pericardium affects cardiac function [29] because the lack of pericardial restrain may influence both global and regional functional parameters. Moreover, there is an increase in artifacts that arise from the myocardium-air interface in MR imaging, that complicate the MR evaluation of open-chest models [15].

The intracoronary ethanol injection model has been described before [18, 36], but to our knowledge, an in-depth MRI and pathological evaluation of the infarcts induced with this technique in the chronic setting is lacking. 

In order to be useful a HF model should have significantly impaired left ventricular function [4], but in this scenario mortality during infarct induction is unacceptably high [7]. The swine mortality in the present study was 16.6%. This mortality rate compares favorably to mortality rates reported for other models, which range between 14% and 67% [4, 6, 7, 33]. Doyle et al. [7] stated that the use of the LAD for infarct induction by 90 min balloon occlusion produced large infarcts in pigs, but it was associated with prohibitively high mortality rates of up to 50% of the animals, and therefore in their paper investigating the effect of balloon inflation during stem cell infusion in an animal model of AMI they used the circumflex artery for infarct induction [7], and none of the six pigs used in their acute study (48-hour followup after AMI) died. Similarly, Reffelmann et al. [33] attempted to perform their MI model in the LAD, but 4/4 animals died, so they opted for the circumflex artery, where still they report a mortality of 52% (11 out of 21 pigs). Stemming from the work of these and other groups, swine are considered to be highly susceptible to malignant arrhythmia development, which increases the mortality rate and complicates the protocols [3, 5, 6]. The low mortality rates encountered in the present study (16.6%) may be related to the use of preoperative antiarrhythmic therapy with amiodarone, which has been suspected to lower the defibrillation threshold [28]. The use of this drug has been also suspected to decrease infarct sizes. Even if this is the case, the percentage of infarcted myocardium in the present study, albeit lower than that reported in other models [17, 18, 20, 28], caused a sustained decrease in LVEF and increase in ventricular volumes over time, with less associated mortality than that encountered in those other studies.

Cardiac MRI allows for quantitative analysis of LV mass and infarct area and has shown high reproducibility in detecting small changes in global and regional mass. It is considered a well validated standard for myocardial function measurement, and its ability to assess chronic infarction by using delayed enhancement has also been established [37]. Reports of MRI-calculated LVEF in healthy swine of any weight are scarce. For this reason, in the present study, as previously performed by Dubois et al. [25], a healthy weight matched group of swine have been subjected to MR studies to determine normal physiologic values in our institution using the same breed, and performed always by the same operator.

As shown in Table 1, LVEF in the present model consistently decreased over time. from a baseline value of 53.8% ± 6.3% prior to model induction to 43.8% ± 7.7% immediately after it, progressing to 46.5% ± 10.1%, 44.5% ± 7.8% and 40.5% ± 56.4% at 7, 30, and 90 days after creation, respectively. In Sasano's model [9], LVEF as measured by echocardiography, showed a similar pattern to ours, from a preoperative value of 68% ± 3% to 57% ± 6% one week after infarction; finally setting in 42% ± 2% at 3 and 5 weeks after the procedure. This behavior probably reflects the initial decrement in EF from the acute event, and a subsequent further decrease from LV remodeling [9], up to the 90 day timepoint, suggesting increasing impairment of cardiac function and heart failure development over time. Similar results have been reported by other groups [6, 25], but these authors, as many others, did not prolong their followup. During the limited study time, our results are comparable to those obtained by Suzuki et al. [6] when occluding the LAD below the origin of the first diagonal branch for 60 minutes. They report an EF of 45.7% ± 6.5% at one week that increased slightly, but not significantly, to 46.9% ± 11.7% at 30 days. In our case, the trend in this parameter between 7 and 30 days was to decrease, but also not significantly. Similarly, Dubois et al. [25], despite not reporting their baseline LVEF value, state that this parameter decreased significantly 1 week after AMI, when it was 46% ± 8%. Again, these investigators describe a nonsignificant increase at 7 weeks, when LVEF was 48% ± 7%. We conducted a longer followup in our model, and LVEF continued to decrease up to the 90day timepoint, suggesting increased impairment of cardiac function and heart failure development. Similar results were obtained in Tomita et al. study [34], where LVEF decreased from a baseline value of 50% ± 5% to 43% ± 4% at 4 weeks and to 37% at 8 weeks, and this decrease in EF was accompanied by LV dilatation and remodeling, as shown by the increased EDV.

A myocardial insult is associated with left ventricular remodeling including changes in geometry, structure, and function [3]. These changes, although initially adaptative, ultimately precipitate the progression of HF. In Dubois et al. study [25], LV remodeling after myocardial infarction was characterized by a marked ESV and EDV expansion, which exceeded natural growth-related increases greater than 2-fold. These authors report a 4% increase in ESV from 1 to 7 weeks of followup in healthy controls, while in infarcted animals it increased more than twice these values. Similarly, EDV increased 26% in healthy controls and 31% in infarcted animals over the same time period. In our study, ESV increased 29.7% from baseline to day 30 and 38.1% from baseline to day 90 in infarcted animals, while these increases in healthy swine were much smaller (9.8% increase from baseline to day 30 and 10.3% increase from baseline to day 90); and EDV in the same time periods changed 17.6% and 28.9% in infarcted animals and 8.8% and 17.6% in healthy controls. It appears clear that in these models, LV dilatation exceeds volume changes related to normal swine growth and aging, thus confirming their value as HF models. 

In the early stages of MI, infarct size can be overestimated by the addition of edema fluid and cellular components, such as hemorrhage and inflammation, which can acutely increase infarct volume by as much as 25% [23, 37]. As this edema and inflammation are healed and necrotic myocardium is replaced by scar tissue over time, the infarcted area shrinks [6]. Researchers must understand this fact to more accurately interpret the results of therapeutic interventions being evaluated. Coupled with the increase in mass secondary to edema and inflammation [37] seen on the acute study, this could explain the apparently large decrease in infarct size evidenced during followup in this study, as been also reported in prior experimental works [6, 25]. 

It is known that remodeling is an ongoing process, so follow-up periods of 6 weeks or shorter have been considered too short to adequately represent the chronic state of the disease in humans [13]. In order to better characterize the model, we aimed for the longer follow-up possible, considering the animals weight gain in our institution and the dimensions of the bore of the MR magnet, which needs to accommodate the animals in the same position in all follow up examinations. 

A thin surviving rim of endocardium, as seen in our model, has been described previously in human transmural infarcts [9]. It has been shown that radiofrequency ablation disrupts ventricular tachycardia circuits by destroying myocardial tissue in this spared subendocardial area [38], a fact that renders our model useful for the study of or training in electrophysiological procedures. 

No model is exempt from weaknesses [3, 16]. The term myocardial infarction refers to myocardial necrosis in a clinical setting consistent of myocardial ischemia [14]. In our model, the heart damage was not secondary to ischemia, but to a chemical injury. Despite the mechanism not being the same, the imaging appearance of these infarcts at 30 and 90 days is representative of fibrosis secondary to infarction. Moreover, akinesia of the affected area is found since very early, and troponin I shows the typical rise and fall pattern required for the diagnosis of acute MI. Another potential limitation is reflected by the fact that the animals grow. In order to minimize the influence of weight gain on our interpretation of the results, we used weight matched controls, as previously reported by Dubois et al. [25]. Lastly, as in all disease modeling, and despite swine being the most representative species, healthy young animals are generally used, while the typical profile of a HF human patient includes middle aged to elderly people suffering from associated cardiovascular problems and risk factors, such as hypertension, atherosclerosis, and diabetes. 

## 5. Conclusions

We have developed a swine model of chronic myocardial infarction by intracoronary ethanol administrations and exhaustively characterized its evolution over 3 months using MRI. This technique consistently results in a transmural infarct, with a mortality analogous to those obtained with other techniques. On the chronic state (90 days), MR and pathology show an image similar to that obtained in the clinical setting. The model may thus be useful for testing therapeutic approaches or new devices in chronic heart failure, such as regenerative or interventional therapies or electrophysiologic procedures, as well as for training specialist in these fields in a setting relevant to the clinical scenario. 

## Supplementary Material

Representative CMR cine sequence obtained in the four-chamber view two hours after intracoronary ethanol administration in swine. Note the akinesia of the septum secondary to the injection.

## Figures and Tables

**Table 1:** Cardiac parameters calculated from magnetic resonance exams performed through the study.

	Baseline	2 hours after induction	Day 7	Day 30		Day 90	
				Study group	Control	Study group	Control
Animal weight (kg)	41.33 ± 11.43	41.33 ± 11.43	42.06 ± 11.70	46.80 ± 5.26	43.20 ± 2.77	62.60 ± 8.76	62.20 ± 6.79
EDV (mL)	62.50 ± 10.36	68.21 ± 9.01	74.30 ± 13.08*	79.57 ± 19.54*	68.46 ± 6.74	99.80 ± 29.70*	75.94 ± 8.47
ESV (mL)	28.65 ± 5.15	38.27 ± 7.02*	39.62 ± 9.18*	45.32 ± 16.66*	31.68 ± 6.79	60.69 ± 22.97^∗†^	31.91 ± 5.46
EF (%)	53.8 ± 6.32	43.79 ± 7.72*	46.54 ± 10.11*	44.48 ± 7.76^∗†^	54.04 ± 5.81	40.48 ± 6.40^∗†^	57.58 ± 8.58
Wall mass (g)	56.67 ± 17.54	64.35 ± 20.62	68.48 ± 18.43	62.46 ± 11.68	57.92 ± 4.42	83.73 ± 17.59	88.68 ± 14.47
CO (L/min)	2.88 ± 0.58	2.63 ± 0.68	2.85 ± 0.79	2.55 ± 0.59	3.32 ± 0.46	2.74 ± 0.79	2.78 ± 0.68
% Infarct MR	n/a	18.09 ± 7.26	12.58 ± 4.10	9.90 ± 5.68	n/a	10.20 ± 4.21	n/a

Data presented as mean ± standard deviation. EDV: end diastolic volume; ESV: end systolic volume; EF: ejection fraction; CO: cardiac output; MR: magnetic resonance; n/a: not applicable. *indicates the existence of statistically significant differences with baseline values (*P* < 0.05). ^†^indicates the existence of statistically significant differences with weight matched controls (*P* < 0.05).
